# Synthesis of Benzene Tetracarboxamide Polyamine and Its Effect on Epoxy Resin Properties

**DOI:** 10.3390/polym10070782

**Published:** 2018-07-16

**Authors:** Seoyoon Yu, Wonjoo Lee, Bongkuk Seo, Chung-Sun Lim

**Affiliations:** Advanced Industrial Chemistry Research Center, Korea Research Institute of Chemical Technology, 45, Jongga-ro, Yugok dong, Jung-gu, Ulsan 44412, Korea; hyeonmin@krict.re.kr (S.Y.); winston@krict.re.kr (W.L.)

**Keywords:** benzene tetracarboxamide polyamine, epoxy resin composition, thermal properties, mechanical properties

## Abstract

Epoxy resins have found various industrial applications in high-performance thermosetting resins, high-performance composites, electronic-packaging materials, adhesives, protective coatings, etc., due to their outstanding performance, including high toughness, high-temperature performance, chemical and environmental resistance, versatile processability and adhesive properties. However, cured epoxy resins are very brittle, which limits their applications. In this work, we attempted to enhance the toughness of cured epoxy resins by introducing benzene tetracarboxamide polyamine (BTCP), synthesized from pyromellitic dianhydride (PMDA) and diamines in *N*-methyl-2-pyrrolidone (NMP) solvent. During this reaction, increased viscosity and formation of amic acid could be confirmed. The chemical reactions were monitored and evidenced using ^1^H-NMR spectroscopy, FT-IR spectroscopy, water gel-phase chromatography (GPC) analysis, amine value determination and acid value determination. We also studied the effect of additives on thermomechanical properties using differential scanning calorimetry (DSC), thermogravimetric analysis (TGA), dynamical mechanical analysis (DMA), thermomechanical analysis (TMA) and by measuring mechanical properties. The BTCP-containing epoxy resin exhibited high mechanical strength and adhesion strength proportional to the amount of BTCP. Furthermore, field-emission scanning electron microscopy images were obtained for examining the cross-sectional morphology changes of the epoxy resin specimens with varying amounts of BTCP.

## 1. Introduction

Epoxy resins are widely used as industrial thermosetting plastics in composites, electronics, adhesives and coatings because of their low price, high toughness, high heat resistance, chemical resistance and excellent mechanical properties [1,2,3,4,5,6,7,8,9,10,11,12,13]. The mechanical properties of epoxy polymers depend on the types of epoxy resins, curing agents and the curing conditions. The presence of multiple amine functional groups in amine curing agents leads to a high crosslinking density during reaction with diglycidyl ether to form polymers which, in turn, causes brittleness, resulting in crack propagation upon external impact and low toughness related to low impact strength.

Therefore, the enhancement of toughness and the mechanical and thermal properties of epoxy polymers has been studied by adding soft polymers, such as amine-terminated butadiene-co-acrylonitrile (ATBN), carboxyl-terminated butadiene-co-acrylonitrile (CTBN), epoxidized natural rubber (ENR), hydroxyl-terminated butadiene-co-acrylonitrile (HTBN), polyethersulfone (PES), poly(etherimide) (PEI), poly(acrylonitrile-butadiene-styrene) (ABS), etc. [14,15,16,17,18,19,20,21,22,23]. While terminal reactive groups of both CTBN and ATBN react with epoxy groups to form a polymer matrix, their flexible rubbery parts are distributed over the matrix to form phase-separated microstructures. The rubbery microstructures act as impact absorber, resulting in an improvement in toughness, while slightly reducing the modulus of the polymer. In addition, engineering thermoplastics like PES, PEI or ABS, with high toughness, ductility and thermally-stable properties are used to enhance the toughness of the epoxy matrix. Elastomers are known to increase the toughness of epoxy polymers, while lowering strength, heat stability and the modulus of the matrix [24].

Herein we prepared a new amine hardener polymer called benzene tetracarboxamide polyamine (BTCP), to compensate for the brittleness of epoxy polymers comprising rigid amine hardeners, like dicyandiamide (DICY) and bisphenol A epoxy resin. We added variable amounts of BTCP (0–20 parts per hundred resin (phr)) in the epoxy compositions and measured the effects on mechanical and thermal properties of the epoxy resin. Furthermore, the fractured surface morphology of the thermosetting polymers was observed using field-emission scanning electron microscopy (FE-SEM).

## 2. Materials and Methods

### 2.1. Materials

**Figure 1:**
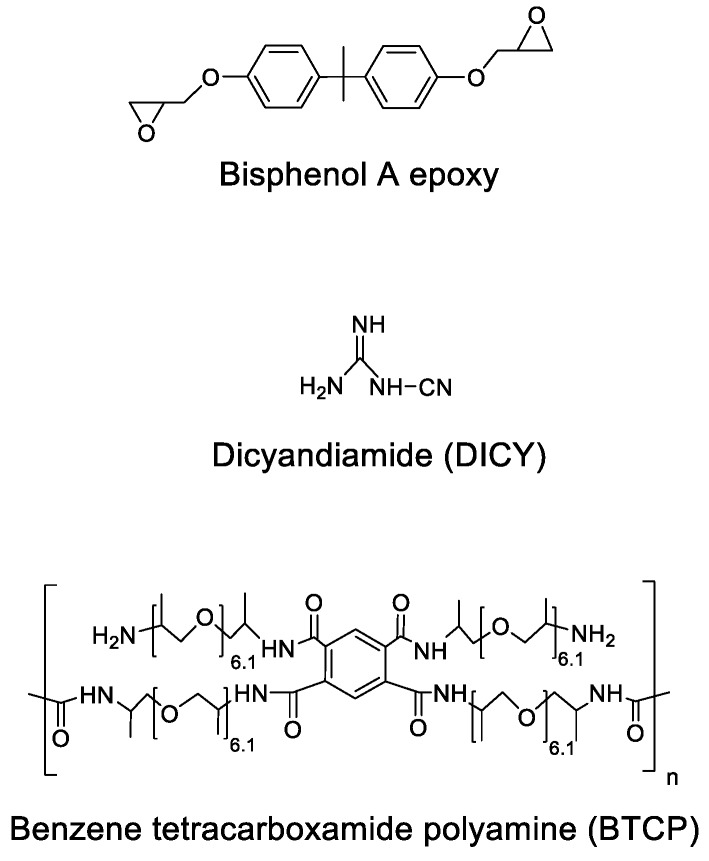
Chemical structures of the epoxy resin (Bisphenol A), curing agent (DICY) and BTCP.

Pyromellitic dianhydride (PMDA) was purchased from TCI (Tokyo, Japan), and *N*-methyl-2-pyrrolidone (NMP) was obtained from Sigma-Aldrich Co. (St. Louis, MO, USA). Liquid Jeffamine D-400 (amine hydrogen equivalent weight (AHEW) = 115 g/eq) was provided by Huntsman (Houston, TX, USA). Diglycidylether of bisphenol A (DGEBA, EPIKOTE 828, epoxy equivalent weight (EEW) = 187 g/eq) was obtained from Momentive Co. (Waterford, NY, USA). Latent curing agent, dicyandiamide (DICY, DICYANEX 1400F) and a urea-based reaction promoter (Amicure UR 7/10) were purchased from Air Products (Allentown, PA, USA). The structures of the epoxy resin, DICY, and BTCP are shown in Figure 1.

### 2.2. Synthesis of Benzene Tetracarboxamide Polyamine (BTCP)

**Figure 2:**
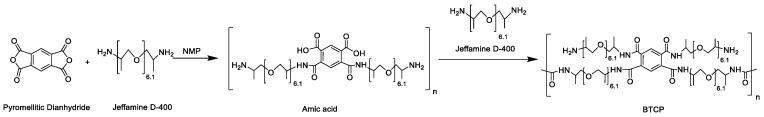
Reaction scheme for the synthesis of BTCP.

The reaction scheme for the preparation of BTCP is shown in Figure 2. Jeffamine D-400 (39.4 g, 91.7 mmol) was dissolved in NMP (60 mL) and the solution was added to a three-neck flask (500 mL) under N_2_ gas. PMDA (10.0 g, 45.8 mmol) was slowly added to the flask and stirred for 30 min to prepare amic acid at room temperature. A small amount of amic acid was obtained for acid value measurement using Equation (1). Based on the calculation of acid value, three equivalents of Jeffamine D-400 were added to the flask and stirred for 1 h at 100–130 °C. After the solution was cooled, NMP was removed by vacuum evaporation. The obtained solid was dried for 6 h at 80 °C. The solid BTCP was processed into fine powder using a freezer mill for use as an associative curing agent for epoxy compositions.

### 2.3. The Preparation of Epoxy Composition and its Curing Process

The detailed formulation ratios of epoxy resin, curing agent and BTCP are shown in Table 1. The epoxy resin was stirred in a 300 mL reactor equipped with a mechanical overhead stirrer for 20 min at 90 °C at 400 rpm. After removal of air bubbles under vacuum, DICY (16.9 g, EEW = 21.0), BTCP (0 to 20 phr) and Amicure (0.31 g) were added and stirred for 20 min to prepare for the epoxy composition.

**Figure 3:**
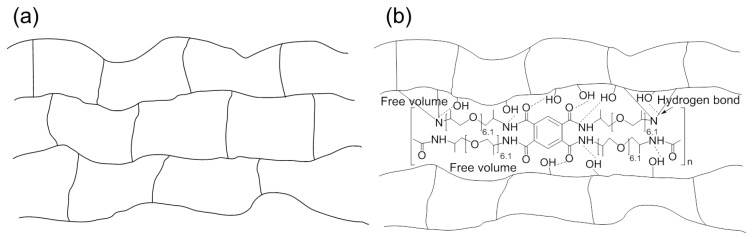
Schematic illustrations of (**a**) neat epoxy networks and (**b**) hydrogen bonds and free volume in BTCP-modified epoxy networks.

The prepared epoxy composition was placed in a metallic mold and heated at 150 °C for 1 h followed by heating at 170 °C for 1 h and at 190 °C for 1 h for measuring thermal and mechanical properties. The cured epoxy polymer network structure and the BTCP-modified polymer network are shown in Figure 3a,b.

### 2.4. BTCP Characterization

The structure of amic acid was confirmed using ^1^H-NMR spectroscopy (300 MHz, Bruker Advanced spectrophotometer, Billerica, MA, USA) at room temperature. The sample was dissolved in DMSO-*d*_6_ with trimethylsilane (TMS) as the internal standard. Furthermore, FT-IR spectrophotometry (Thermo Nicolet 6700, Thermo Scientific, Waltham, MA, USA) was used to observe the change in functional groups of both reactants and products. Moreover, the acid value of the amic acid was calculated using Equation (1). Acid value is the number of milligrams of KOH required to neutralize the acid in 1 g of sample. The mass of Jeffamine D-400 required to neutralize amic acid is determined by titrating amic acid.

(1) $$AV=\frac{V_{\mathrm{EP}1}\times f\times c\left(\mathrm{KOH}\right)\times M_{A}}{m_{s}}$$

where *AV* is the acid value (mg KOH/g), $V_{\mathrm{EP}1}$ is the volume (mL) of titrant *c*(KOH), f is a factor, $M_{A}$ is the molecular weight of KOH (56.11 g/mol) and $m_{s}$ is the sample weight (g). The molecular weight of BTCP was measured using gel-phase chromatography (GPC, Agilent, Santa Clara, CA, USA) with water eluent at room temperature, while the melting temperature was confirmed by using the M-565 device (BUCHI, Flawil, Switzerland).

### 2.5. Analysis of Mechanical and Thermal Properties of the Cured Epoxy Compositions

Thermal information, such as the onset temperature (*T*_onset_), of epoxy compositions was collected using differential scanning calorimetry (DSC, TA instruments Q2000, New Castle, DE, USA) performed in the temperature range of 25–300 °C at a heating rate of 10 °C. The glass transition temperature (*T*_g_) was measured by the second DSC run. Dynamic mechanical analysis (DMA) to measure viscoelastic properties was performed on a Q800 instrument (TA instruments, New Castle, DE, USA). The processed test specimens of dimensions 60 mm × 12 mm × 3 mm were mounted on the dual cantilever probe. The test was performed in the temperature range of 30–250 °C at a heating rate of 5 °C/min, frequency of 1 Hz, and amplitude of 10 µm to obtain the storage modulus, loss modulus and tan *δ*. Thermomechanical analysis (TMA, Q500, TA instruments, New Castle, DE, USA) was used to analyze the linear coefficient of thermal expansion (CTE) and the *T*_g_ of cured epoxy polymers processed to dimensions of 5 mm × 5 mm × 3 mm. The test specimens were placed on the expansion-type probe followed by an increase in temperature by 2 °C/min from 30 to 250 °C. The effects of different amounts of BTCP in epoxy compositions on the mechanical properties of the epoxy resin were studied by measuring the flexural, tensile and impact strengths. The tensile strength was measured using the ASTM D-638 method by employing a universal testing machine (UTM, model 5982, INSTRON, Norwood, MA, USA). The test specimen was processed to dimensions of 150 mm × 13 mm × 3 mm. The test was repeated five times to obtain an averaged value. The flexural strength was measured using the ASTM D 790M method by employing using UTM with test specimens having dimensions of 60 mm × 25 mm × 3 mm. The test was repeated five times to obtain an averaged value. The impact strength was measured with an Izode pendulum impact tester (JJHBT-6501, JJ-test, Chengde, Hebei, China) by the ASTM D256 method. Test samples were processed to dimensions of 63.5 mm × 12.7 mm × 3 mm with a 2.54 mm notch. Five cured polymers were tested for each epoxy composition to obtain an averaged value.

### 2.6. Morphology Analysis

The fractured surface of the cured epoxy plastic obtained from the impact test was observed using FE-SEM (MIRA 3, Tescan Co., Brno-Kohoutovice, Czech Republic) after coating with Pt.

## 3. Results

### 3.1. Synthesis of BTCP 

The reaction products of both amic acid and BTCP were analyzed with ^1^H-NMR, FT-IR and measurement of the acid value.

**Figure 4:**
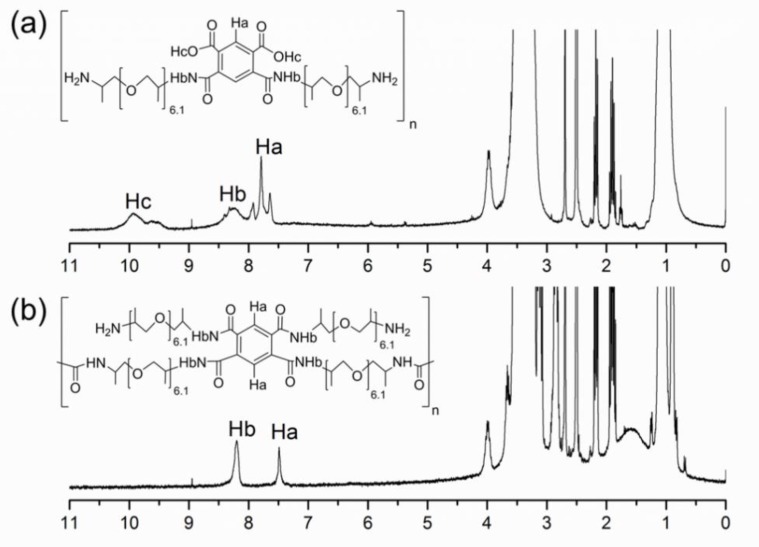
^1^H-NMR spectra of (**a**) amic acid and (**b**) BTCP.

Amic acid: ^1^H-NMR (300 MHz, DMSO-*d*_6_) (Figure 4a): *δ* (ppm) = 10.3–9.4 (2H, s), 8.7–8.1 (2H, s), 8.0–7.5 (2H, m).

BTCP: ^1^H-NMR (300 MHz, DMSO-*d*_6_) (Figure 4b): *δ* (ppm) = 8.6–8.0 (4H, m), 7.3–7.6 (H, s), 7.6–8.0 (H, s).

Figure 4a shows the ^1^H-NMR spectra of amic acid, while Figure 4b shows the ^1^H-NMR spectra of BTCP. The signal for the carboxylic proton of amic acid (Figure 4a) at 10.3–9.4 ppm is absent in Figure 4b, owing to the formation of amide by the reaction between acid and amine. This reaction is also confirmed by the 1:2 integral ratio of H_a_ against H_b_.

**Figure 5:**
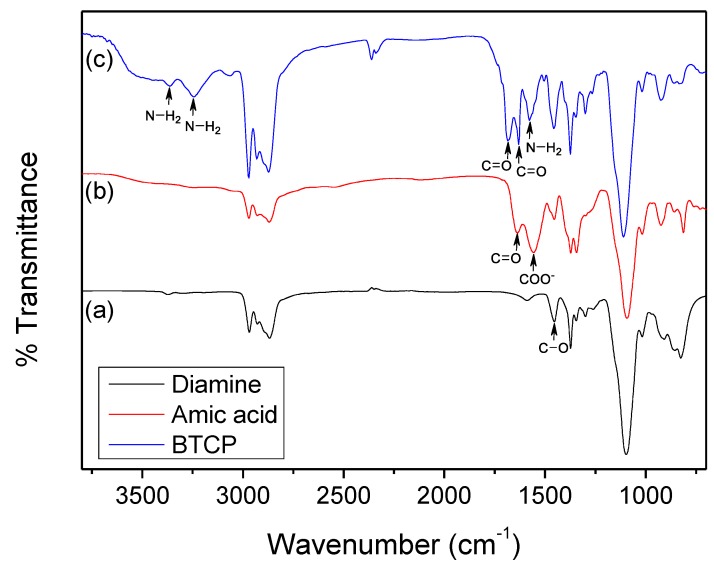
FT-IR spectra of (**a**) diamine, (**b**) amic acid and (**c**) BTCP.

FT-IR was performed before and after the reaction, and the results are shown in Figure 5. The C=O stretching peak of amic acid observed at 1625 cm^−1^ (Figure 5b) shifted to 1670 cm^−1^ owing to the formation of the amide group of BTCP (Figure 5c). The antisymmetric stretching peak (COO–) also shifted from 1560 cm^−1^ for amic acid to 1625 cm^−1^ for BTCP. In addition, there are NH_2_ stretching peaks at 3220 cm^−1^ and 3360 cm^−1^, as well as an NH_2_ bending peak at 1590 cm^−1^ (Figure 5c).

The reaction from amic acid to BTCP was followed by measurements of the acid value using Equation (1); the acid value decreased dramatically from 0.070 g KOH/g for amic acid to 0.019 g KOH/g for the reaction product to form BTCP.

The molecular weight of the produced BTCP was measured by gel permeation chromatography (GPC), and the obtained data are provided in Table 2. The measured number average molecular weight (*M_n_*) was 463,200 g/mol, and the weight average molecular weight (*M_w_*) was 572,640 g/mol. The measured melting point of BTCP was 190 °C.

### 3.2. Thermal and Mechanical Properties of Epoxy/Prepared Amine Curer Compositions

Thermal data including the onset temperature, *T*_peak_ and *T*_g_ obtained from the DSC experiments are presented in Table 3. The onset temperature and *T*_peak_ are proportional with the amount of BTCP, while *T*_g_ is inversely proportional to the amount of BTCP. The *T*_g_ of neat epoxy observed at 125.81 °C decreased to 108.74 °C when the amount of BTCP in the epoxy composition reached 20 phr. The addition of BTCP led to a slight decrease in *T*_g_, which is attributed to an increase in the amount of dissolved BTCP within the epoxy matrix. This behavior was also observed in other rubber- or elastomer-modified epoxy systems [25,26,27].

**Figure 6:**
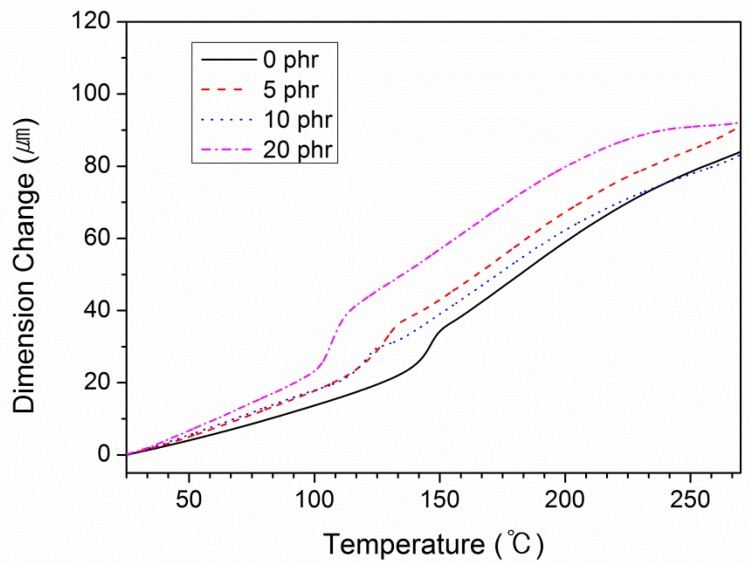
TMA graphs of epoxy compositions.

Figure 6 shows the results of the thermomechanical analysis (TMA) of the compositions, the data for which are presented in Table 4. The linear coefficient of thermal expansion (CTE) is obtained around *T*_g_. *α*_1_ represents a CTE below *T*_g_ and *α*_2_ implies a CTE above *T*_g_. The CTE value of *α*_1_ tends to increase with the amount of BTCP, whereas the CTE of *α*_2_ decreases with increasing amount of BTCP. In general, when the CTE value increases, the internal stress also increases, causing a crack in the polymer matrix, which, in turn, decreases the adhesive strength. Therefore, as other studies have also shown, it is preferable to lower the CTE value [28].

**Figure 7:**
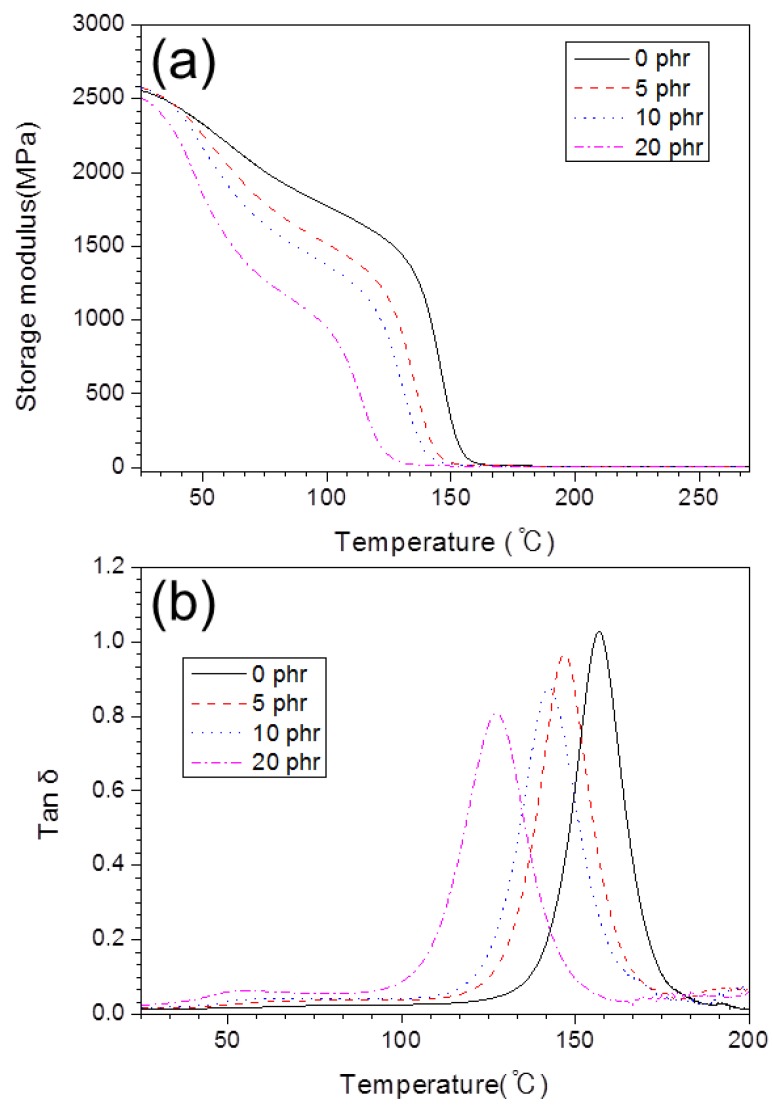
DMA graphs of epoxy compositions (**a**) storage modulus and (**b**) tan *δ*.

The dynamic mechanical analysis (DMA) data, including the values of tan *δ* and the storage modulus (*E*’) of each epoxy composition, are shown in Figure 7 and listed in Table 5. We examined the behavior of the cured polymers over a wide range of temperatures, in low-(25–30 °C), intermediate-(50–100 °C), and high-temperature regions (100–200 °C). In the low-temperature region, all epoxy composites show similar values of the storage modulus (Figure 7a). In general, the storage modulus values (*E*’) of the epoxy compositions including BTCP were lower than that of the neat epoxy composition system. A sharp decrease in the storage modulus was obtained in all epoxy compositions near *T*_g_. The temperature at which the sharp decrease begins tends to decrease in proportion to the amount of BTCP. The decrease of tan *δ* follows a similar trend to that shown in Figure 7b. This suggests that the rubbery properties of BTCP lower both the storage modulus (*E*’) and tan *δ* of the epoxy compositions. The height of tan *δ* for the epoxy compositions, including BTCP, is lower than that of the neat epoxy composition, because of the size decrease in the epoxy phase resulting from the increase of the BTCP content, which forms the rubber-rich phase [29].

The polymer chains of the epoxy composition with 20 phr of BTCP (sample 4) started to move at a lower temperature, as compared to the neat epoxy resin. As the fluidity of the polymer chain is inversely proportional to the crosslinking density, the fluidity of the polymer chains decreases as the crosslinking density increases [30]. In fact, neat epoxy, with a relatively high crosslinking density, shows a higher *T*_g_ than that of sample 4. Therefore, the epoxy compositions with BTCP are predicted to compensate for the brittleness of the epoxy polymer due to the lower crosslinking density.

**Figure 8:**
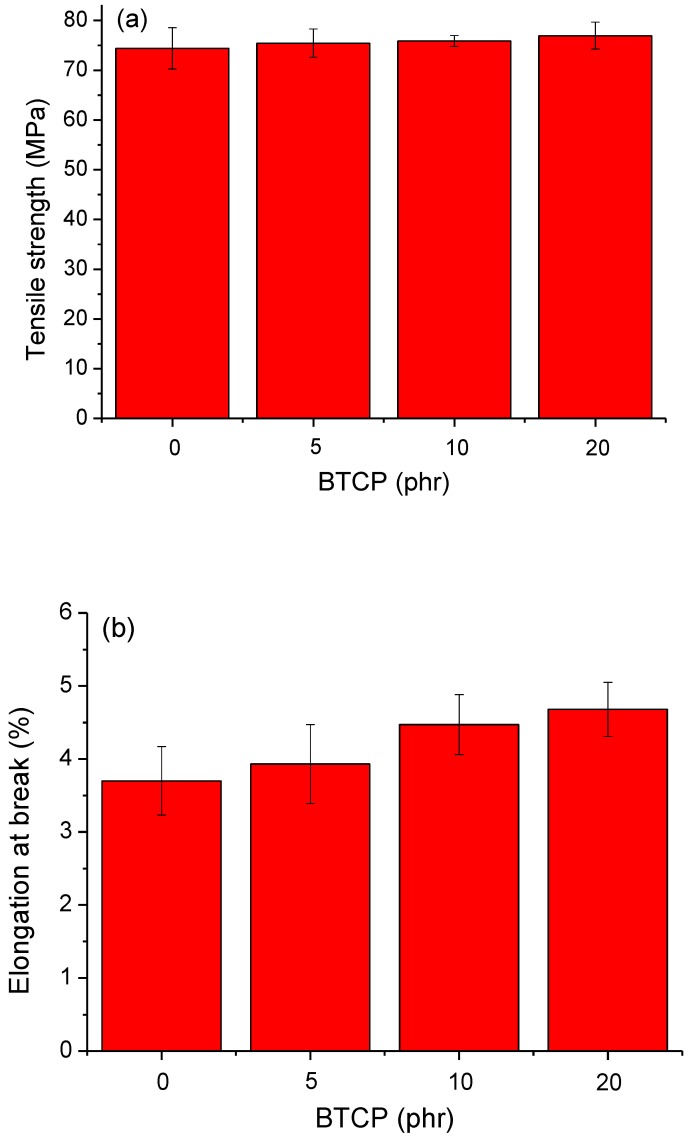
Universal tensile testing: (**a**) tensile strength and (**b**) elongation at break of cured epoxy compositions.

**Figure 9:**
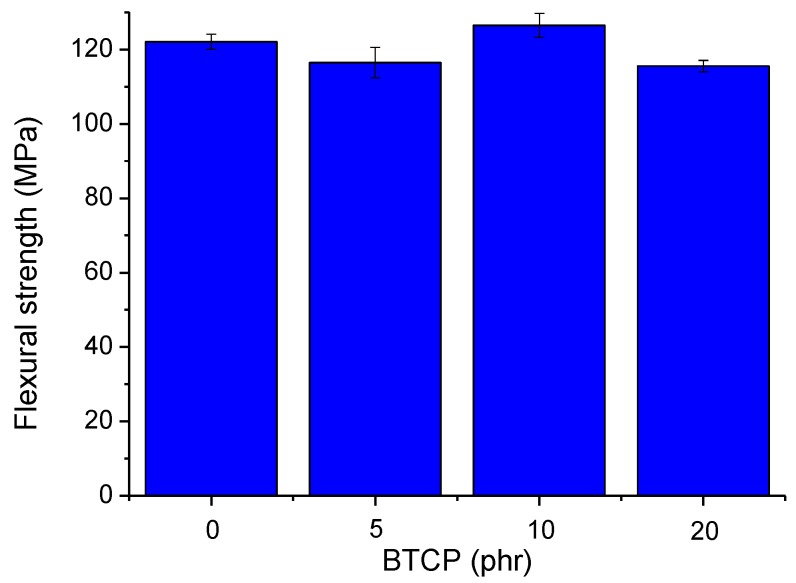
Flexural strength of cured epoxy samples.

The mechanical properties of the cured epoxy compositions were analyzed by measuring their tensile strength, flexural strength, and Izod impact strength. Figure 8a,b show the results of the tensile strength tests. The tensile strength (Figure 8a) of each epoxy composition increases, from 74.40 MPa for neat epoxy to 76.96 MPa for sample 4, with increasing amounts of BTCP. This slight improvement in the tensile strength can be explained by the improved hydrogen bonding in the polymer (Figure 3b) after curing the epoxy polymer [31]. Additionally, the primary amines of BTCP react with the glycidyl ether of the epoxy resin to participate in the epoxy polymer network. The cured epoxy compositions with BTCP exhibit a more flexible and elastic behavior during loading than the neat epoxy composition. The elongation of the cured epoxy compositions with 20 phr of BTCP shows an increase of 26.5% from 3.7% to 4.68%. (Figure 8b). Typically, the addition of rubbery tougheners to the polymer matrix decreases the tensile strength. Akbari et al. [32] and Saleh et al. [13] mentioned a decrease in the tensile strength of CTBN-modified epoxy resin. Moreover, Thomas et al. [33] reported a decrease in the tensile strength of epoxy resin modified with hydroxyl-terminated polybutadiene (HTPB) liquid rubber. Notably, one benefit of the BTCP used here is that it does not seriously deteriorate the physical performance of the epoxy polymers. Flexural strength data in Figure 9 show that the addition of flexural BTCP does not result in a loss of flexural strength, as compared to that in a neat epoxy composition, unlike other toughening agents, which decrease the flexural strength [13,33].

**Figure 10:**
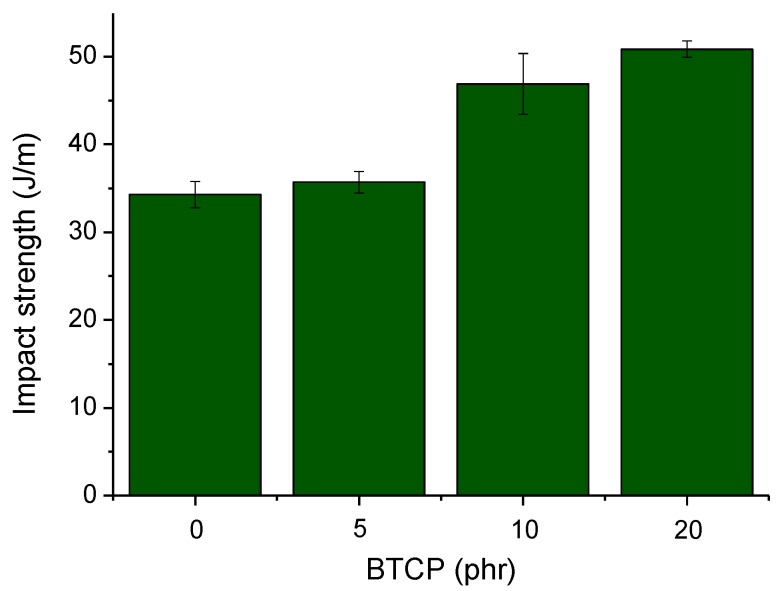
Impact strength of cured epoxy samples.

Figure 10 shows the Izod impact strength of the notched epoxy composition test specimen as a function of the BTCP content. The impact strength of epoxy compositions with BTCP is significantly greater than that of the neat epoxy composition. The impact strength of the neat epoxy composition increased by approximately 48% by adding 20 phr of BTCP, from 34.28 J/m to 50.84 J/m (Figure 10). The increment in the Izod impact strength of the epoxy composition can be attributed to the strong interaction, as well as good compatibility, between the epoxy matrix and BTCP. This suggests that BTCP is useful to toughen polymers for enhancing the impact resistance, while maintaining other physical properties.

### 3.3. FE-SEM Analysis

**Figure 11:**
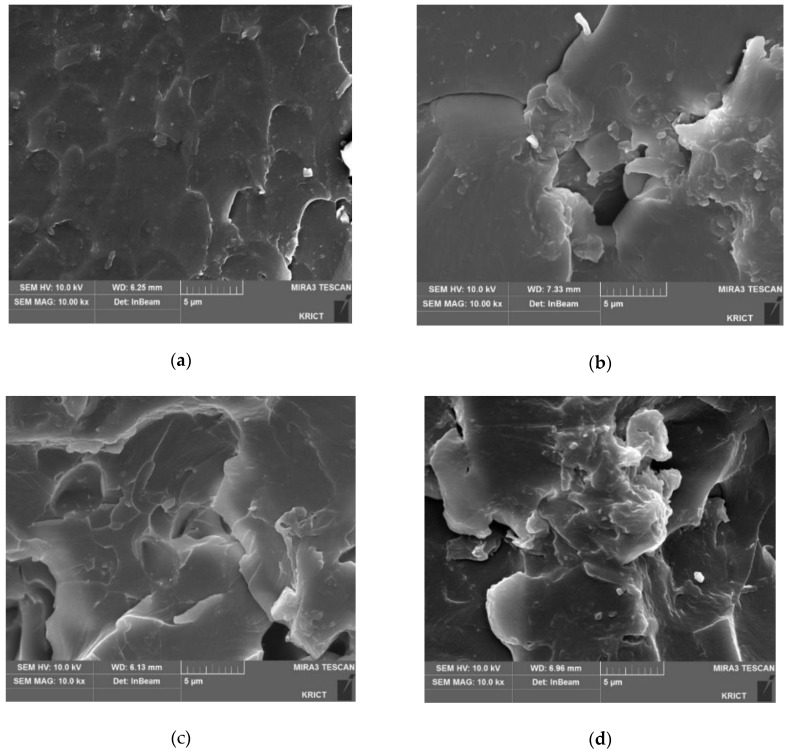
FE-SEM images of the fractured surface after impact testing with different BTCP contents: (**a**) 0 phr, (**b**) 5 phr, (**c**) 10 phr and (**d**) 20 phr.

Figure 11 shows the FE-SEM images of the test specimen obtained after measuring the impact strength. The fracture morphology of the cured neat epoxy resin composition (Figure 10a) looks smooth, and shows a slight wavy pattern caused by the brittleness of epoxy resin. As the content of BTCP increases from 5 phr to 20 phr, the area torn out from the fracture surface increases, which is an indication of enhanced toughness. The increase in roughness is caused by the distortion of intramolecular cavities (free space) when the specimen undergoes the impact strength test. For 20% of BTCP added to the composition, the fractured surface is the roughest, and the impact strength is 48.3% higher than that of the cured neat epoxy. In addition, all samples exhibit homogeneous morphology without the presence of a second phase.

## 4. Conclusions

The structure of BTCP was determined by GPC, FT-IR, ^1^H-NMR and acid value measurements. The tensile strength of the epoxy compositions increased with increasing amounts of BTCP, from 74.4 MPa for neat epoxy to 76.96 MPa for the composition with 20 phr BTCP.

Although most toughening agents with rubbery parts reduce the flexural strength, BTCP in varying contents in the compositions did not deteriorate the flexural strength. Moreover, the impact strength of the compositions increased by 48.3% with the amount of BTCP, from 34.28 J/m for neat epoxy to 50.84 J/m for sample 4. In addition, FE-SEM images showed that the fracture surface in the modified system had a rough and irregular appearance. The degree of roughness also increased with the amount of additive BTCP. Therefore, based on the results of the impact strength and FE-SEM experiments, BTCP is promising as an epoxy toughener.

## Figures and Tables

**Table 1:** Formulations of epoxy compositions.

Sample	BTCP (phr)	Epoxy (g)	DICY (g)	Amicure (g)	BTCP (g)
1	0	150	16.86	0.315	0
2	5	150	16.86	0.315	7.5
3	10	150	16.86	0.315	15
4	20	150	16.86	0.315	30

**Table 2:** Characterization of BTCP.

Appearance	White Powder
Acid value (mg KOH g^−1^)	0.019
*M_n_* (g/mol)	463,200
*M_w_* (g/mol)	572,630
*M_w_*/*M_n_*	1.23
Melting temperature (°C)	190

**Table 3:** DSC data of epoxy/BTCP compositions.

Sample	BTCP (phr)	Onset temperature (°C)	*T*_peak_ (°C)	*T*_g_ (°C)
1	0	154.55	169.74	125.81
2	5	155.11	169.96	119.36
3	10	157.93	1·72.29	117.49
4	20	163.45	175.82	108.74

**Table 4:** TMA data for each epoxy composition with varying amounts of BTCP.

Sample	BTCP (phr)	CTE (μm/m·°C)		*T*_g_ (°C)
		*α*_1_	*α*_2_	
1	0	62.27	125.20	146.47
2	5	79.80	111.20	132.02
3	10	80.13	100.80	120.68
4	20	106.30	71.98	107.06

**Table 5:** DMA data of epoxy compositions.

Sample	BTCP (phr)	Storage modulus (MPa)		Tan *δ*
		25 °C	175 °C	
1	0	2553	12.79	156.76
2	5	2574	11.52	146.84
3	10	2574	10.90	142.07
4	20	2505	9.28	127.07
